# 18F-Fluorodeoxyglucose Uptake in Abdominal Aortic Aneurysms: A Useful Biomarker of AAA Rupture Risk

**DOI:** 10.1155/2014/930738

**Published:** 2014-09-18

**Authors:** Kosmas I. Paraskevas, Dimitri P. Mikhailidis, Frank J. Veith

**Affiliations:** ^1^Sheffield Vascular Institute, Northern General Hospital, Sheffield S5 7AU, UK; ^2^Department of Clinical Biochemistry (Vascular Disease Prevention Clinics), University College London Medical School, Royal Free Hospital Campus, University College London (UCL), London NW3 2QG, UK; ^3^Divisions of Vascular Surgery, New York University Langone Medical Center, New York, NY 10016, USA; ^4^Division of Vascular Surgery, The Cleveland Clinic, Cleveland, OH 44106, USA

Moris et al. discuss several emerging biomarkers implicated in the pathophysiology of abdominal aortic aneurysms (AAAs) [1]. These include biomarkers related to AAA extracellular matrix homeostasis or proteolysis, cellular or signalling pathways, proteins released by intraluminal thrombi, circulating cells and inflammation, metabolomics and genetic biomarkers. One emerging biomarker which probably also deserves to be mentioned is 18F-fluorodeoxyglucose (18F-FDG).

18F-FDG uptake detected by positron emission tomography (PET) is used to assess hypermetabolic activity of cells in tumors and inflammatory processes [2, 3]. Inflammation plays a key role in the development of AAAs [4]. AAA regions displaying increased 18F-FDG uptake show increased inflammatory activity and are enriched in leukocytes [5]. Increased 18F-FDG uptake in AAAs is associated with inflammation, aortic wall instability, and rupture risk [6]. Therefore, 18F-FDG uptake might be a new technique to study AAA disease in vivo and may improve prediction of AAA rupture risk [6, 7]. The prognostic value of 18F-FDG uptake was verified by a study reporting increased focal uptake of 18F-FDG in patients with large, rapidly expanding, or symptomatic AAAs that are prone to rupture [8].

In a recent study [9], biopsies of the AAA wall were obtained from regions with no 18F-FDG uptake and from regions positive for 18F-FDG uptake, both at the site positive for uptake and at a distant negative site of the AAA wall. The sites with a positive 18F-FDG uptake were characterized by a higher number of adventitial inflammatory cells and by a reduction of smooth muscle cells in the media compared with the negative 18F-FDG samples [9]. It was concluded that positive 18F-FDG uptake in the AAA wall is associated with an active inflammatory process and alterations of the expression of genes involved in the remodelling of adventitia and collagen degradation, which potentially participate in the weakening of the AAA wall preceding rupture [9]. Another use of 18F-FDG may be in endovascular AAA repair (EVAR) procedures [10]. Increased 18F-FDG uptake following EVAR may be an indirect predictor of AAA sac enlargement due to the presence of an endoleak (even if this is not detected by imaging modalities) and increased AAA rupture risk [10].

18F-FDG uptake may be a more promising biomarker for AAAs than some of those discussed by the authors (e.g., cystatin C and neutrophil gelatinase-associated lipocalin [NGAL]) [1]. There is evidence that NGAL and cystatin C are affected by kidney function, as well as by statins [11–13]. In turn, patients with AAAs may have impaired kidney function and may well be on statins [14]. To make things even more complicated, statins can improve renal function in vascular patients [15]. These factors make the use of these markers subject to confounding.

In conclusion, 18F-FDG may prove to be a useful biomarker in the pathogenesis of AAAs. Future studies should investigate these possibilities.
